# Impact of (poly)phenol-rich dietary sources on DNA damage: insights from human intervention studies using the Comet assay – a review and perspective

**DOI:** 10.1017/S000711452500073X

**Published:** 2025-07-14

**Authors:** Misko Milev, Boris Roglev, Maria Kondeva Rogleva, Milena Georgieva, George Miloshev, Tatjana Ruskovska

**Affiliations:** 1 Faculty of Medical Sciences, Goce Delcev University, Stip, North Macedonia; 2 Diaverum Clinic, Gevgelija, North Macedonia; 3 Laboratory of Molecular Genetics, Epigenetics and Longevity, Institute of Molecular Biology “Roumen Tsanev”, Bulgarian Academy of Sciences, Sofia, Bulgaria

**Keywords:** Single-cell gel electrophoresis, Oxidative DNA damage, Polyphenol, Chronic non-communicable diseases, Cardiovascular, Cardiometabolic, Neurodegenerative, Cancer

## Abstract

(Poly)phenols are plant-derived food bioactives abundantly present in human diet. They exert positive effects on various aspects of human health and in particular in reducing the risk of chronic non-communicable diseases. Dietary (poly)phenols have been reported to improve vascular function, blood lipids, insulin sensitivity and to decrease systemic inflammation. Evidence also suggests that (poly)phenols may exert protective effects on DNA, by reducing the extent of its damage. In recent years, advanced analytical methods, including transcriptomics, metabolomics, proteomics and metagenomics, have been employed to unravel the complex impact of (poly)phenols in health and disease. Advances in bioinformatics enable an integrated multi-omics approach to data analysis, opening avenues for discovering new, previously unknown molecular mechanisms of action. Innovative solutions and automation of the Comet assay offer new opportunities for more in-depth analysis of the impact of (poly)phenols on DNA damage and its inclusion in integrative bioinformatic models. Such an approach has the potential to uncover new multi-level interactions and to reveal previously unknown factors underlying inter-individual variabilities in health-promoting effects of (poly)phenols. This review provides an insight into the application of the Comet assay in human intervention studies using (poly)phenol-rich dietary sources. Recent advancements in the Comet assay technology and the prospects for more extensive use of this method in future human intervention studies with (poly)phenols could contribute to the development of personalized dietary recommendations for these plant-derived food bioactives.

## Graphical abstract

**Figure:**
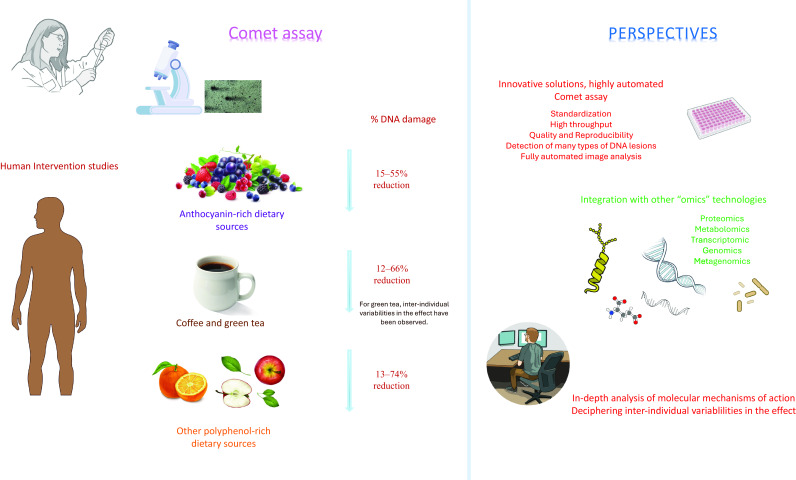

Chronic non-communicable diseases, such as cardiometabolic and neurodegenerative diseases, as well as cancer, are highly prevalent and leading causes of morbidity and mortality^(1–3)^. These diseases are present with distinct clinical manifestations and are managed with specific clinical interventions, but at the molecular level, they share several common mechanisms. Recent evidence indicates that inflammation is an important common pathophysiological trait in cardiometabolic, neurodegenerative diseases and cancer^(4–7)^, and that aberrant inflammasome activation can cause uncontrolled tissue responses, potentially contributing to these diseases^(8)^. Chronic low-grade inflammation is closely associated with disturbed cellular redox status, i.e. an imbalance between oxidants and antioxidants, which, if unresolved, leads to oxidative stress^(9,10)^, another common mechanism underlying the chronic non-communicable diseases and ageing^(11)^. Oxidative stress causes significant damage to biomolecules such as lipids, proteins and DNA^(12)^, and consequently, triggers profound disturbances in cellular functions. Several assays have been developed to measure the origine and extent of oxidative stress, some of which may even have potential clinical relevance^(13)^. For example, to determine the level of oxidative DNA damage, two methods are most commonly used: (a) the quantification of urinary excretion of the nucleoside 8-oxo-7,8-dihydro-2′-deoxyguanosine^(14)^ and (b) single-cell gel electrophoresis, also known as the Comet assay.

The Comet assay is a rapid, sensitive, versatile and affordable method for measuring DNA damage in eukaryotic cells. In this assay, cells are embedded in low melting point agarose on a microscopic slide and lysed to disrupt nuclear membrane and unpack to a certain extent the DNA in the chromatin. In this process, the DNA remains attached to the nuclear matrix and lamina at different intervals, forming supercoiled loops in a structure known as a nucleoid. In the presence of DNA strand breaks, supercoiling is relaxed, and the DNA loops migrate towards the anode upon application of electrophoresis, creating the characteristic ‘Comet’ tail. Undamaged DNA remains in the head of the ‘Comet’. The extent of DNA migration towards the anode correlates with the severity of DNA damage in the cell.

The Comet assay was first introduced 40 years ago as a method for detecting DNA damage at the level of individual cells^(15)^. In 1988, the method was further modified and optimised to use alkaline conditions, which convert alkali-labile sites into DNA strand breaks, thereby increasing the specificity and reproducibility of this assay^(16)^. Later, an additional step involving the digestion of DNA with lesion-specific enzymes was introduced. This step converts specific lesions into DNA breaks, increasing the intensity of the Comet tail. This modification marked a new era in the Comet assay, enhancing its sensitivity and allowing it to differentiate between various types of DNA damage. The first enzyme used was the endonuclease III, which recognises oxidised pyrimidines^(17)^. Subsequently, formamidopyrimidine DNA glycosylase (Fpg) was introduced to detect oxidised purines^(18)^. While several other enzymes have also been employed, Fpg and endonuclease III remain the most widely used for human biomonitoring purposes^(19)^. Modifications of the Comet assay, which involve subjecting cells to various challenges, such as hydrogen peroxide or iron (III) chloride^(20)^ to assess cellular resistance to oxidative stress or benzo[*a*]pyrene^(21)^ to evaluate resistance to genotoxicity, are also widely used.

The most commonly used visualisation method involves staining the DNA with a fluorescent dye and analysing it under fluorescence microscopy, therefore allowing a high degree of automatisation^(22)^. An alternative silver staining method is also available but not widely used due to its high labour requirements. The advantages of the silver staining method include its low cost, the long-term preservation of slides, reduced hazard risks and the ability to perform the analysis using a simple light microscope^(23)^.

(Poly)phenols are secondary plant metabolites with various functions, including protection against herbivores and pathogenic microorganisms, attraction of pollinators and seed-dispersing animals, protection from UV irradiation or playing a role as signalling molecules in the formation of nitrogen-fixing root nodules^(24)^. More than 9000 different (poly)phenols have been identified in plants, of which only several hundred are relevant to human nutrition. Dietary (poly)phenols are classified into two major groups: flavonoids and non-flavonoids. Flavonoids are the most extensively studied and are further divided into several classes: anthocyanins, chalcones, dihydrochalcones, dihydroflavonols, flavanols, flavanones, flavones, flavonols and isoflavonoids. The non-flavonoid group includes lignans, phenolic acids, stilbenes and other (poly)phenols^(25)^. The daily intake of dietary (poly)phenols varies across populations^(26,27)^, but it is generally accepted that the average intake is approximately 1 g of total (poly)phenols per day^(28,29)^.

Studies have reported a plethora of health-promoting properties of (poly)phenols, although those are often not associated with total (poly)phenols, but rather with specific (poly)phenol (sub)class/es. Beneficial health effects of (poly)phenols include (a) a decreased risk of diabetes, cardiovascular events, and all-cause mortality^(28)^; (b) the improvement of cognitive impairment associated with neurodegenerative disorders^(30)^ and (c) promising effects to decrease the risk of cancer^(31)^. Importantly, a recent large-scale, randomised, double-blind, placebo-controlled study conducted among 21 442 USA adults (12 666 women aged ≥65 years and 8776 men aged ≥60 years), all free of major cardiovascular disease and recently diagnosed cancer, randomly assigned to either a flavanol-rich cocoa extract supplement [500 mg flavanols/day, including 80 mg (–)-epicatechin] or a placebo, showed that the cocoa extract supplementation reduced the cardiovascular disease death by 27 %^(32)^. Around the same time, the *First Ever Dietary Bioactive Guideline* was published, recommending a daily intake of 400–600 mg of flavan-3-ol, for cardiometabolic protection^(33)^. Despite this significant progress in the field of (poly)phenols and human health, many aspects still require further exploration to find adequate solutions, such as a) developing guidelines for other (poly)phenol (sub)classes; b) addressing the inter-individual variabilities of their health effects^(34)^ and c) developing personalised intake recommendations as an ultimate future goal.

The molecular mechanisms underlying the beneficial health effects of (poly)phenols have been extensively studied, particularly in recent years, with the use of advanced analytical and bioinformatic technologies, such as omics, multi-omics and integrative bioinformatics^(35,36)^. These studies have the potential to identify specific genetic polymorphisms for future nutrigenetic studies, which could lead to a better understanding of inter-individual variabilities in the health effects of (poly)phenols^(37,38)^. Additionally, these studies have shown that common molecular mechanisms of action of (poly)phenols involve cellular processes such as cell adhesion and mobility, immune system, metabolism or cell signalling, as well as several cellular pathways involved in the inflammation^(37)^.

Nuclear factor erythroid 2-related factor 2 (NRF2)-mediated antioxidant defence has also been identified as one of the molecular mechanisms by which (poly)phenols may exert their cardiometabolic protective effects^(36)^. Indeed, numerous studies have demonstrated the positive effects of (poly)phenols on oxidative DNA damage^(39)^. However, animal studies often use very high, pharmacological concentrations and non-oral routes of administration, which are not relevant to human nutrition. Similarly, *in vitro* studies are often conducted with extracts and/or bioactive compounds that do not appear in the circulation after the processes of absorption, distribution, metabolism and excretion, i.e. in physiologically irrelevant experimental conditions. Therefore, in this study, we focused on human intervention studies with dietary (poly)phenols at quantities relevant to human nutrition, aiming to answer the question: What is the evidence of protective effects of dietary (poly)phenols on DNA damage in humans, as demonstrated using the Comet assay? To this end, we conducted a systematic literature search and detail our findings in this review paper.

## Literature search

Our literature search was registered in the PROSPERO database under registration number CRD42020162357. The registration date in PROSPERO was 28 April 2020, and the record was updated on 10 February 2023^(40)^. The literature search was performed on PubMed, with no restrictions on publication date. Only papers published in English were considered for inclusion in this review.

Within the context of oxidative cell damage, which is relevant for cardiometabolic and neurodegenerative diseases or cancer, this literature search was focused on DNA damage assessed with the Comet assay in any cell type, and DNA-protective and antioxidant properties of nutritional (poly)phenols. According to the study protocol, studies involving healthy individuals or patients with cardiometabolic and neurodegenerative diseases or cancer, both men and women, were considered for inclusion in this review. Studies focused on adolescents (under 18 years of age) or elderly people (over 70 years of age) were not considered. This review includes human intervention studies with dietary (poly)phenols at quantities relevant to human nutrition. These include pure compounds, extracts or foods and beverages rich in (poly)phenols. Interventions with medicinal plants were not considered.

On 15 September 2019, a literature search was conducted on PubMed using the following search terms: (polyphenol OR flavonoid) AND (comet OR genotoxicity). This search yielded a total of 1026 scientific papers. The papers were screened for the use of the Comet assay in human intervention studies with (poly)phenols, retrieving seventeen potentially eligible studies. According to the study protocol, two reviewers independently screened the records. In cases of disagreement, a third reviewer was consulted. A follow-up search using the same search terms was conducted on PubMed on 31 January 2023, to identify any eligible human intervention studies published after the initial search; however, no additional studies were retrieved.

**Figure 1.**
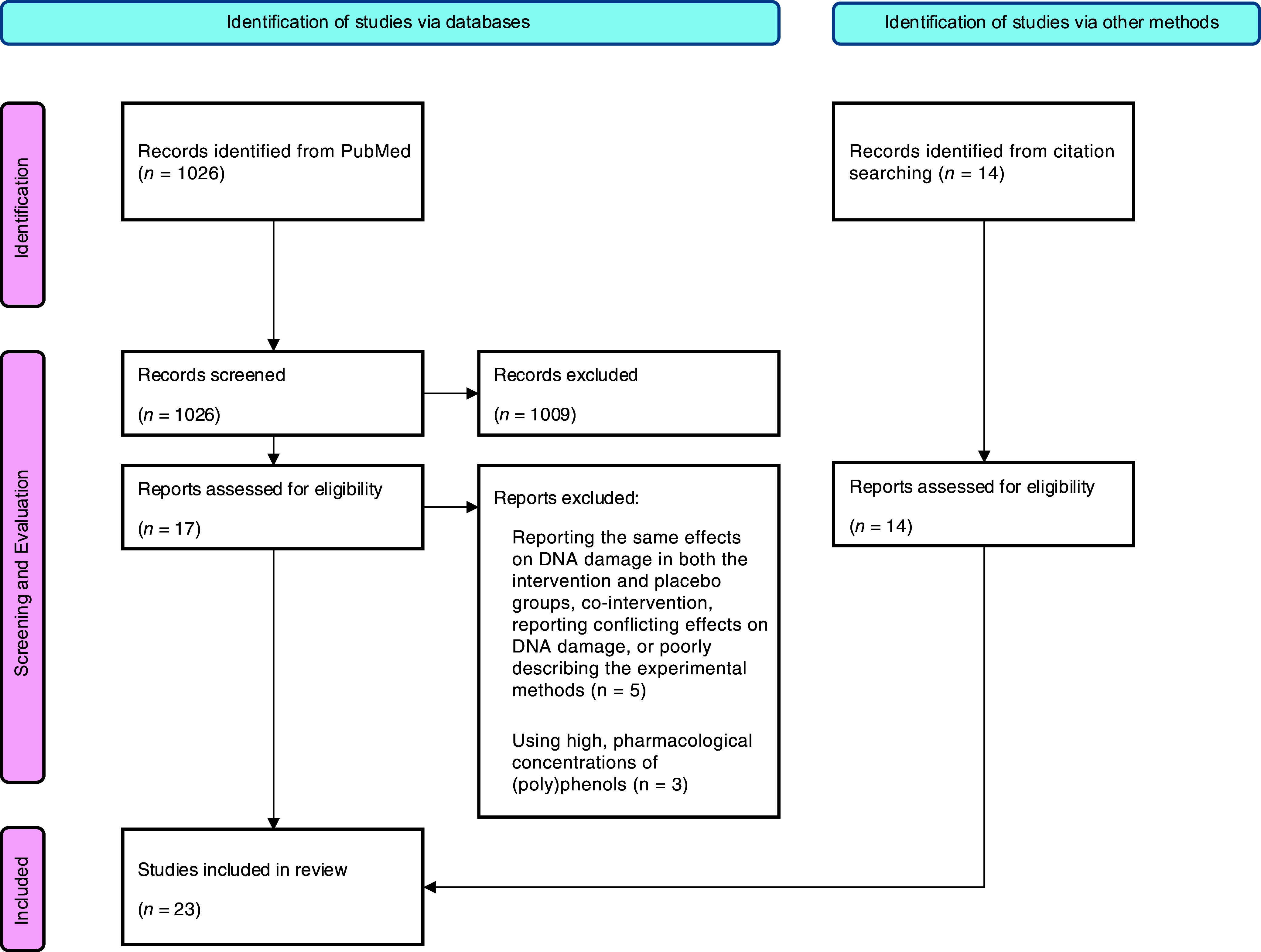
Workflow of the literature search.

During the evaluation process, five studies were excluded for the following reasons: reporting the same effects on DNA damage in both the intervention and placebo groups, co-intervention with a carotenoid, reporting conflicting effects on DNA damage or poorly describing the experimental methods. Additionally, three more studies were excluded due to the use of high, pharmacological concentrations of (poly)phenols, which are not relevant for human nutrition. However, during the evaluation process, fourteen additional studies were identified and subsequently included in this review. Ultimately, a total of twenty three human intervention studies were included, with only two reporting an upper age range above 70 years. Nonetheless, since these two studies involved participants with diseases – namely, haemodialysis patients^(41)^ and those with type 2 diabetes^(42)^ – we decided to include them in our review. The workflow of the literature search is presented in Fig. 1, using a flow diagram adapted from Preferred Reporting Items for Systematic reviews and Meta-Analyses 2020 statement^(43)^.

Following the screening and evaluation, one reviewer extracted the data, and another checked the extracted data. Again, in cases of disagreement, a third reviewer was consulted. The extracted data were included in an Excel table specifically designed for this literature search. The data included: information about the paper (PMID, authors, title and year of publication); whether the study focused on cardiometabolic disease, neurodegenerative disease or cancer; positive outcomes other than oxidative stress parameters; study design; number, age and sex of participants; health status of participants; type of Comet assay; type of cells analysed with the Comet assay; (poly)phenol used for treatment; dose; placebo; duration of the treatment; Comet assay outcomes; other genotoxicity assays (if conducted); outcomes of the other genotoxicity assays (if applicable); oxidative stress parameters, other than Comet assay (if conducted) and outcomes of these oxidative stress parameters (if applicable).

Data extracted from the eligible human intervention studies (*n* 23) were further evaluated and the studies categorised into four groups based on the (poly)phenol-rich food, beverages or plant extract under study: (1) anthocyanin-rich food and beverages, (2) coffee, (3) green tea and (4) others. The results are presented in online Supplemental Table 1. Selected data from the online Supplemental Table 1 are presented in Table 1. Additionally, numerical data extracted or estimated from studies reporting statistically significant positive outcomes of the Comet assay following the consumption of (poly)phenol-rich dietary sources are presented in Table 2.

**Table 1.** Human intervention studies on the impact of (poly)phenol-rich dietary sources on DNA damage assessed using the Comet assay

Reference; PMID	Study design	Study population				Type of Comet assay	Sample	(Poly)phenol or (poly)phenol-rich food/beverage	Dose	Outcome of the Comet assay
		Number	Age	Sex	Health status					
^(44)^ 23507228	Randomised, placebo-controlled, cross-over study	10	20·8 ± 1·6	Male	Healthy, non-smokers	1. Comet assay with Fpg enzyme (oxidised purines) 2. H_2_O_2_-induced DNA damage	Blood mononuclear cells	Blueberries	One portion (300 g) of blueberries	Decreased H_2_O_2_-induced DNA damage 1 h after blueberry intake. Oxidised purines evaluated through quantification of Fpg-sensitive sites were not significantly different.
^(45)^ 22733001	Randomised, repeated-measures crossover study	18	47·8 ± 9·7	Male	Healthy, with at least one risk factor for CVD	1. Comet assay with Fpg enzyme (oxidised purines) 2. H_2_O_2_-induced DNA damage	Blood mononuclear cells	Wild blueberry (WB) drink	25 g of WB freeze-dried powder, providing 375 mg of anthocyanins, once per day	Decreased oxidised purines evaluated through quantification of Fpg-sensitive sites. Decreased H_2_O_2_-induced DNA damage.
^(46)^ 16892265	First study, 18 participants, 9-week intervention study with flavonoid/anthocyanin-rich mixed fruit juice. Weeks 1–2 (run-in), weeks 3–6 (juice uptake) and weeks 7–9 (wash-out). Second study conducted with identical design, but with a control juice and 9 participants.	18	27·7 ± 4·9	Male	Healthy, non-smokers	1. Comet assay with Fpg enzyme (total DNA damage) 2. Comet assay without Fpg enzyme (basal DNA damage)	Whole blood	Red mixed fruit juice produced from red grape juice (57 %), blackberry juice (18 %), sour cherry juice (9 %), black currant juice (9 %) and chokeberry juice (7 %).	700 ml juice/day in three equal portions TEAC = 19·1 mmol/l Trolox	Highly significant decrease of total DNA damage. Basal DNA damage was significantly lowered.
^(47)^ 12943921	Intervention study	67	19–57	51 Male 16 Female	Healthy, both smokers and non-smokers	1. Alkaline Comet assay	Lymphocytes	Purple grape juice	480 ml juice/day in two portions	Significantly decreased endogenous DNA damage.
^(48)^ 12667600	Randomised crossover study	27	35 ± 4	Male	Healthy non-smokers	1. Alkaline Comet assay 2. Comet assay with endonuclease III (oxidised pyrimidines)	Peripheral blood mononuclear cells	Juice A rich in anthocyanin-providing aronia, blueberries and boysenberries. Juice B contained flavanol-rich green tea, apricot and lime.	330 ml/day consumed with main dishes	Decreased oxidised pyrimidines in the second supplementation period. No effect on single-strand breaks.
^(49)^ 16032375	Randomised controlled study	20	18–40	Female	Healthy, mostly non-smokers	1. Alkaline Comet assay 2. Comet assay with endonuclease III (oxidised pyrimidines) 3. H_2_O_2_-induced DNA damage	Lymphocytes	Cranberry juice	750 ml/day (3×250 ml)	No significant effects on DNA damage.
^(50)^ 15225586	Controlled parallel intervention study	57	19–52	20 Male 37 Female	Healthy, mostly non-smokers	1. Alkaline Comet assay 2. Comet assay with Fpg enzyme 3. Comet assay with endonuclease III	Mononuclear blood cells	Blackcurrant juice Anthocyanin drink	Daily doses ranged from 475 to 1000 ml according to body weight, ingested during three daily meals	Increase of Fpg-sensitive sites within the blackcurrant juice group suggesting even a possible adverse effect.
^(41)^ 19064553	Pilot intervention study (3 weeks run-in, 4 weeks juice uptake, and 3 weeks wash-out)	21	21–79	14 Male 7 Female	Clinically stable haemodialysis patients	1. Comet assay with Fpg enzyme (total DNA damage) 2. Comet assay without Fpg enzyme (basal DNA damage)	Whole blood	Red mixed fruit juice produced from red grape juice (40 %), blackberry juice (20 %), sour cherry juice (15 %), black currant juice (15 %), and elderberry juice (10 %).	200 ml juice/day in two equal (100 ml) portions	Highly significant decrease of total DNA damage. Basal DNA damage was not significantly different.
^(51)^ 27016493	Intervention study	25	66·2 ± 2·6	15 Male 10 Female	Haemodialysis patients	1. Comet assay with Fpg enzyme (oxidative DNA damage) 2. Comet assay without Fpg enzyme (basal DNA damage)	Lymphocytes	Unfermented grape juice	100 ml juice three times per week, during the last half hour of haemodialysis session	Significant decrease of oxidative DNA damage. Nonsignificant effect on basal DNA damage.
^(52)^ 16099480	Human intervention study (the third trial)	7	26·0 ± 6·0	not reported	Healthy non-smokers	1. (+/–)-anti-benzo[*a*]pyrene-7,8-dihydrodiol-9,10-epoxide-induced DNA-damage	Lymphocytes	Unfiltered coffee	1 litre/day	Highly significant decrease of (+/–)-anti-benzo[*a*]pyrene-7,8-dihydrodiol-9,10-epoxide-induced DNA-damage.
^(53)^ 17376579	Human intervention study	8	20–50	not reported	Healthy non-smokers	1. Comet assay under standard conditions 2. Comet assay with Fpg enzyme (oxidised purines) 3. Comet assay with endonuclease III (oxidised pyrimidines) 4. H_2_O_2_-induced DNA damage 5. Trp-P-2-induced DNA damage	Lymphocytes	Filtered coffee (paper filtered and metal filtered)	600 ml (400 ml paper filtered and 200 ml metal filtered)/d	Decreased oxidised purines. Decreased oxidised pyrimidines. Decreased H_2_O_2_-induced DNA damage. Decreased Trp-P-2-induced DNA damage. Nonsignificant Comet assay under standard conditions.
^(54)^ 20709087	Controlled intervention study with a cross-over design	38	27·6 ± 8·0	14 Male 24 Female	Healthy non-smokers	1. Comet assay under standard conditions 2. Comet assay with Fpg enzyme (oxidised purines) 3. Comet assay with endonuclease III (oxidised pyrimidines) 4. H_2_O_2_-induced DNA damage	Lymphocytes	Paper filtered coffee	800 ml coffee/day, without a fixed daily schedule	Decreased oxidised purines. Other types of Comet assay were nonsignificant.
^(55)^ 21462335	Human intervention study	33	20–44	Male	Healthy non-smokers	1. Alkaline Comet assay without Fpg enzyme 2. Alkaline Comet assay with Fpg enzyme	White blood cells	Freshly brewed coffee rich in both green and roast bean coffee constituents	750 ml/day in three equal portions (morning, noontime, afternoon)	Marked decrease of DNA damage –Fpg. Marked decrease of DNA damage +Fpg.
^(56)^ 26632023	Short-term repeated uptake human intervention study	13	20–50	Male	Healthy non-smokers	1. Alkaline Comet assay	White blood cells	Freshly brewed coffee rich in both green and roast bean coffee constituents	800 ml in four equal portions (4×200 ml) every two hours	Significant reduction of background DNA strand breaks.
^(57)^ 24740588	Prospective, randomized, controlled study with parallel design	84 in total 42 in the coffee group 42 in the control group	19–50	Male	Healthy non-smokers	1. Alkaline Comet assay	Whole blood	Freshly brewed coffee rich in both green and roast bean coffee constituents	750 ml coffee/day in three equal portions	Decreased spontaneous DNA strand breaks.
^(58)^ 20589860	Controlled intervention study with a cross-over design	29	20–55	13 Male 16 Female	Healthy non-smokers	1. Comet assay with Fpg enzyme (oxidised purines) 2. Comet assay with endonuclease III (oxidised pyrimidines) 3. H_2_O_2_-induced DNA damage	Lymphocytes	Coffee (instant coffee co-extracted from green and roasted beans)	800 ml coffee/day	No significant effects on DNA damage.
^(59)^ 15634219	Intervention pilot study	10	30–57	4 Male 6 Female	Healthy, non-smokers	1. Irradiation-induced DNA damage	Whole blood	Green tea	540 ml green tea (3 green tea drinks in close succession)	Significantly decreased DNA damage after 12 min exposure to UVA/VIS.
^(60)^ 20452781	Intervention pilot study	9	22–39	3 Male 6 Female	Healthy, non-smokers	1. Irradiation-induced DNA damage	Whole blood	Green tea	540 ml green tea (3 green tea drinks in close succession)	Significantly decreased DNA damage after 12- and 18-min exposure to UVA/VIS, 60 min post green tea in the group of responders.
^(61)^ 17349075	Randomized repeated measures design	7	26·0 ± 2·1	Female	Healthy non-smokers	1. H_2_O_2_-induced DNA damage	Mononuclear blood cells	Blood orange juice	300 ml, single portion	Increased resistance to H_2_O_2_-induced DNA damage.
^(62)^ 17696483	Double-blind, randomized, cross-over study	6	27 ± 3	Male	Healthy non-smokers	1. Alkaline Comet assay 2. Comet assay with Fpg enzyme (oxidised purines) 3. Comet assay with endonuclease III (oxidised pyrimidines) 4. H_2_O_2_-induced DNA damage 5. FeCl_3_-induced DNA damage	Lymphocytes	Apple (organic or conventional)	1000 g/day	24 h after consumption, a statistically significant decrease in the level of endonuclease III-sensitive sites and an increased capacity to protect DNA against damage induced by iron chloride for both conventional and organic apples. Changes in endogenous DNA strand breaks, Fpg-sensitive sites, or capacity to protect DNA against damage caused by hydrogen peroxide were nonsignificant.
^(42)^ 9892240	Randomized crossover study	10	50–74	5 Male 5 Female	Diabetic, type 2 (stable, healthy in other respects)	1. H_2_O_2_-induced DNA damage 2. Comet assay with endonuclease III (oxidised pyrimidines)	Lymphocytes	High-flavonol diet	Low-flavonol diet supplemented with 76–110 mg of flavonols provided by 400 g of onions (and tomato sauce) and six cups of tea daily	Decreased H_2_O_2_-induced DNA damage. No significant effect on oxidised pyrimidines.
^(63)^ 10443336	Randomized controlled study	10 in total soya milk (*n* 4), rice milk (*n* 3), cow’s milk (*n* 3)	20–50	Male	Healthy non-smokers	1. Alkaline Comet assay 2. Comet assay with endonuclease III (oxidised pyrimidines) 3. H_2_O_2_-induced DNA damage	Lymphocytes	Soy milk	1 litre/day	Progressive decrease in oxidised pyrimidines. No significant protective effect against endogenous DNA strand breakage or H_2_O_2_-induced DNA damage.
^(64)^ 22981768	Intervention study	12	30·2 ± 12·8 25·2 ± 3·1	5 Male 7 Female	Healthy, non-smokers	1. Comet assay under standard conditions 2. Comet assay with Fpg enzyme (oxidised purines) 3. H_2_O_2_-induced DNA damage	Lymphocytes	Pills containing: 2 mg resveratrol from grapes, 100 mg dried grape extract, 50 mg dried extract from olive oil, 3 mg lycopene, 100 mg vitamin C and 30 mg bioflavonoids from citrus fruits.	3 pills/day	No significant effects on DNA damage.

**Table 2.** Numerical data extracted or estimated from studies reporting statistically significant positive outcomes of the Comet assay following the consumption of (poly)phenol-rich dietary sources

Reference; PMID	(Poly)phenol-rich food/beverage	Statistically significant positive outcomes of the Comet assay	Measure of DNA damage	Numerical values	% Decrease of DNA damage
Anthocyanin-rich food and beverages					
^(44)^ 23507228	Blueberries	H_2_O_2_-induced DNA damage	% DNA in tail (mean ± sd)	Before the intervention: 51·7 ± 4·9 After the intervention: 42·7 ± 8·7	17·4 %
^(45)^ 22733001	Wild blueberry drink	Oxidised purines H_2_O_2_-induced DNA damage	% DNA in tail (mean ± sd)	Before the intervention: 12·5 ± 5·6 After the intervention: 9·6 ± 3·5 Before the intervention: 45·8 ± 7·9 After the intervention: 37·2 ± 9·1	23·2 % 18·8 %
^(46)^ 16892265	Red mixed fruit juice	Total DNA damage (with Fpg enzyme) Basal DNA damage (without Fpg enzyme)	% Tail intensity (estimated)	Run-in period: ≈2·9 Juice uptake period: ≈1·3Difficult to estimate the % of tail intensity from the figure	≈55 % /
^(47)^ 12943921	Purple grape juice	Endogenous DNA damage	Comet tail length (µm) (mean ± sem)	Smokers Before the intervention: 92·3 ± 2·1 After the intervention: 69·6 ± 2·1 Non-smokers Before the intervention: 86·0 ± 2·1 After the intervention: 70·8 ± 1·7	24·6 % 17·7 %
^(48)^ 12667600	Anthocyanin-rich juice A and flavanol-rich juice B	Oxidised pyrimidines	% Fluorescence in tail (estimated)	Run-in period: ≈46 Second supplementation period: ≈25	≈46 %
^(41)^ 19064553	Red mixed fruit juice	Total DNA damage (with Fpg enzyme)	% Tail intensity (mean)	Run-in period: 5·22 Juice uptake period: 2·85	45·4 %
^(51)^ 27016493	Unfermented grape juice	Oxidative DNA damage (with Fpg enzyme)	% DNA in tail (mean ± se)	Before the intervention: 26·36 ± 1·30 After the intervention: 22·41 ± 1·18	15·0 %
Coffee					
^(52)^ 16099480	Unfiltered coffee	(+/–)-Anti-benzo[*a*]pyrene-7,8-dihydrodiol-9,10-epoxide induced DNA-damage	Comet tail length (µm) (mean ± sd)	Before the intervention: 24·34 ± 4·20 After the intervention: 15·56 ± 4·25	36·1 %
^(53)^ 17376579	Filtered coffee	Oxidised purines Oxidised pyrimidines H_2_O_2_-induced DNA damage Trp-P-2-induced DNA damage	Comet tail length (µm) (mean) Comet tail length (µm) (estimated)	Before the intervention: 3·5 After the intervention: 1·2 Before the intervention: 3·8 After the intervention: 1·9 Before the intervention: ≈32 After the intervention: ≈27 Before the intervention: ≈33 After the intervention: ≈22	65·7 % 50·0 % ≈16 % ≈33 %
^(54)^ 20709087	Filtered coffee	Oxidised purines	% DNA in tail (mean ± sd)	After water consumption: 10·29 ± 3·45 After coffee consumption: 9·02 ± 4·28	12·3 %
^(55)^ 21462335	Freshly brewed coffee	DNA damage – Fpg DNA damage +Fpg	% Tail intensity (estimated)	Before the intervention: ≈1·15 After the intervention: ≈0·70 Before the intervention: ≈5·10 After the intervention: ≈2·85	39 % 44 %
^(56)^ 26632023	Freshly brewed coffee	Background DNA strand breaks	% Tail intensity (mean)	Before the intervention: 0·33 After the intervention: 0·22	33 %
^(57)^ 24740588	Freshly brewed coffee	Spontaneous DNA strand breaks	% Tail intensity (estimated)	Before the intervention: ≈0·32 After the intervention: ≈0·27	≈16 %
Green tea					
^(59)^ 15634219	Green tea	DNA damage after 12-min exposure to UVA/VIS	% DNA in tail (mean)	Before the intervention: 17·07 After the intervention: 11·67	31·6 %
^(60)^ 20452781	Green tea	DNA damage after 12- and 18-min exposure to UVA/VIS, 60 min post green tea in the group of responders	% DNA in tail (estimated)	12 min exposure Before the intervention: ≈ +5·0 After the intervention: ≈ –4·0 18 min exposure Before the intervention: ≈ +4·5 After the intervention: ≈ –4·5	Not reported in the paper and difficult to calculate from the data presented in the relevant figure.
Other					
^(61)^ 17349075	Blood orange juice	H_2_O_2_-induced DNA damage	% DNA in tail (mean ± sd)	Before the intervention: 62·9 ± 6·3 After the intervention: 52·0 ± 14·5	17·3 %
^(62)^ 17696483	Apple (organic or conventional)	Endonuclease III-sensitive sites FeCl_3_-induced DNA damage	% Tail intensity (mean ± sd)	Organic Before the intervention: 3·69 ± 2·61 After the intervention: 1·10 ± 1·20 Conventional Before the intervention: 4·60 ± 1·23 After the intervention: 1·19 ± 0·73 Organic Before the intervention: 9·38 ± 2·89 After the intervention: 5·12 ± 1·67 Conventional Before the intervention: 9·23 ± 3·05 After the intervention: 5·63 ± 1·73	70·2 % 74·1 % 45·4 % 39·0 %
^(42)^ 9892240	High-flavonol diet	H_2_O_2_-induced DNA damage	Arbitrary units of DNA damage (mean ± sd)	Low flavonol diet: 220 ± 12 High flavonol diet: 192 ± 14	12·7 %
^(63)^ 10443336	Soya milk	Oxidised pyrimidines	Arbitrary units of DNA damage (estimated)	Before the intervention: ≈58 After the intervention: ≈19	≈67 %

## Anthocyanin-rich food and beverages

Anthocyanins are water-soluble pigments that give the red, purple and blue colour to plants. The main dietary sources of anthocyanins include berries and fruit-derived beverages. In foods, anthocyanins are present as glycosides. The sugar-free, aglycone forms of anthocyanins are called anthocyanidins. To date, approximately twenty-seven different anthocyanidins have been identified in nature, but only six cyanidin, delphinidin, pelargonidin, peonidin, malvidin and petunidin, are predominantly present in the human diet^(65)^. The beneficial health effects of dietary anthocyanins have been extensively studied and have been summarised in a recent comprehensive review^(66)^. These effects include the attenuation or even mitigation of the development and progression of atherosclerosis, metabolic syndrome and various types of cancer through cellular mechanisms such as increased antioxidative defence, reduced free radical damage or decreased inflammation and risk of mutations^(66)^.

With our literature search, we identified nine studies with anthocyanin-rich food or beverages^(41,44–51)^. Among these studies, only one was conducted with anthocyanin-rich fruit (blueberries) in form of a jelly^(44)^, while the other studies were conducted with different anthocyanin-rich beverages such as wild blueberry drink, red mixed fruit juice, purple grape juice, mixed fruit juice, cranberry juice, blackcurrant juice or unfermented grape juice. Apart from one acute study^(44)^, the other studies had durations ranging from 2 weeks to 6 months.

Regarding the participants’ health status, three studies were conducted with healthy non-smokers^(44,46,48)^, two with healthy participants who were mostly non-smokers^(49,50)^, one with healthy participants who included both smokers and non-smokers^(47)^, one with healthy participants with at least one risk factor for CVD^(45)^ and two with haemodialysis patients^(41,51)^.

Regarding the sex of the participants, four studies included only males^(44–46,48)^, one study included only females^(49)^ and four studies included both males and females^(41,47,50,51)^. All studies used nutritionally relevant doses of anthocyanin-rich fruit jelly or beverages, but only five of them were designed as controlled interventions^(44–46,49,50)^.

Regarding the outcomes of the Comet assay, most of the anthocyanin studies (*n* 7) reported significant improvements in at least one measure of DNA damage. These included decreased H_2_O_2_-induced DNA damage^(44)^, decreased oxidised purines and H_2_O_2_-induced DNA damage^(45)^, a highly significant decrease in total DNA damage and significantly decreased basal DNA damage^(46)^, significantly decreased endogenous DNA damage^(47)^, decreased oxidised pyrimidines^(48)^, a highly significant decrease in total DNA damage^(41)^ and a significant decrease in oxidative DNA damage^(51)^. The reduction in DNA damage reported in these studies ranges from 15 to 55 % (Table 2). One study reported no significant effects^(49)^, while the study by Møller P, *et al.* reported an increase in Fpg-sensitive sites (i.e. an increase in oxidised purines) within the blackcurrant juice group, suggesting a potential adverse effect^(50)^.

Among the genotoxicity assays other than the Comet assay, the micronucleus test was used in only one study^(51)^, but the results were non-significant. On the other hand, in seven out of the nine studies, various parameters related to redox balance or oxidative stress were evaluated. Of these, four studies reported significant improvements in at least one of the evaluated parameters, such as significant improvement in glutathione status^(46)^, significant decrease in plasma total free radicals^(47)^, a decrease in plasma thiobarbituric acid reactive substances^(48)^ or a significant improvement of glutathione status, significant decrease of plasma malondialdehyde and significant decrease of plasma protein carbonyls^(41)^.

## Coffee

Coffee is one of the most popular beverages, widely consumed and enjoyed not only for its stimulating effects on the central nervous system but also for its pleasant taste and aroma. Hundreds of compounds have been identified in coffee, including caffeine and numerous (poly)phenols from the class of phenolic acids. The main phenolic acid in coffee is 5-caffeoylquinic acid (*aka* chlorogenic acid), although other compounds from the same class are also present in significant quantities. Controversies still exist regarding coffee consumption and its effects on human health, but it is generally accepted that ‘for adults consuming moderate amounts of coffee (3–4 cups per day, providing 300–400 mg of caffeine), there is little evidence of health risks and some evidence of health benefits’^(67)^. Notably, in terms of health effects of coffee intake, the genetic background is very important, as some individuals have a reduced capacity to metabolise caffeine, which may lead to adverse health effects when consumed in larger quantities^(68)^.

Interestingly, using our keywords no studies on the effects of coffee consumption on DNA damage were retrieved. This is likely due to the search terms being very general and not specifically including (poly)phenols specific to coffee. However, during the evaluation of other papers already selected for their eligibility, we identified seven human intervention studies with coffee, assessing its protective effects on various measures of DNA damage^(52–58)^. Apart from one acute study^(56)^, all other studies were chronic, lasting between 5 days to four weeks. All studies were conducted with healthy non-smokers. Three of the studies included only males^(55–57)^, two included both males and females^(54,58)^ and two did not report any sex information of the participants^(52,53)^. The doses used for interventions were relatively high, ranging from 600 ml to 1 litre per day, but still within the range that can realistically be consumed by healthy adults. Only three out of the seven studies were controlled, where consumption of equal amounts of water was used as the control for the amount of coffee consumed^(54,57,58)^. Additional methods for evaluating genotoxicity were not considered in any of these studies. However, four studies assessed different aspects of oxidative stress and antioxidant defence. Three of these studies reported statistically significant improvements in specific parameters, such as increased superoxide dismutase activity in cytosolic fractions of lymphocytes^(53)^, increased total and reduced glutathione along with increased glutathione reductase activity^(55)^ and decreased 3-nitrotyrosine and 8-isoprostaglandine F2a^(58)^. Additionally, one study reported statistically significant positive outcomes other than oxidative stress parameters, specifically a decrease in body weight and body fat^(55)^.

Finally, there were statistically significant positive outcomes in at least one measure of DNA damage as assessed by the Comet assay in six out of the seven studies, such as a highly significant decrease in (+/–)-anti-benzo[*a*]pyrene-7,8-dihydrodiol-9,10-epoxide-induced DNA-damage^(52)^, decreased oxidised purines, decreased oxidised pyrimidines, decreased H_2_O_2_-induced DNA damage, decreased 3-amino-1-methyl-5H-pyrido[4,3-*b*]indole acetate-induced DNA damage^(53)^, decreased oxidised purines^(54)^, marked decrease in DNA damage ±Fpg^(55)^, significant reduction of background DNA strand breaks^(56)^ and decreased spontaneous DNA strand breaks^(57)^. The reduction in DNA damage reported in these studies ranges from 12 to 66 % (Table 2). Only one study reported non-significant modulations of DNA damage as a result of coffee consumption^(58)^.

## Green tea

Like coffee, tea is one of the most popular beverages worldwide. Green tea, black tea and oolong tea, all made from the same plant, *Camellia sinensis*, are categorised based on their respective manufacturing processes into non-fermented green tea, semi-fermented oolong tea and fermented black tea. Tea is probably the most popular energising drink with well documented health benefits. Studies suggest that tea consumption is inversely associated with the risk of cardiovascular disease. Notably, a recent umbrella review of systematic reviews concluded that ‘it is reasonable to judge that 2 cups of unsweet tea per day has the potential to decrease cardiovascular disease risk and progression due to its flavonoid content’^(69)^. Green tea is a major dietary source of flavonoids, particularly flavan-3-ols, which include (−)-epigallocatechin 3-*O*-gallate, (−)-epigallocatechin, (−)-epicatechin 3-*O*-gallate and (−)-epicatechin. Beneficial health effects of green tea consumption, beyond reducing cardiovascular disease risk, include anticancer activity, anti-obesity and antidiabetic effects, neuroprotective effect or gut health-promoting properties^(70)^.

Surprisingly, in our literature search, we only identified two human intervention studies investigating the effects of green tea on DNA damage. These studies assessed the protective effect of green tea against UVA/VIS-induced DNA damage, which is relevant for skin cancer^(59,60)^. Both studies were acute and conducted over a duration of 40 or 90 min after the participants consumed the final cup of tea. The studies were designed as pilot intervention studies, involving a small number of healthy non-smokers of both sexes who were asked to drink 540 ml green tea (3 teacups in a row). These studies did not include any control treatment. Other parameters for genotoxicity and/or redox status were not assessed.

Both studies showed a protective effect of green tea consumption on UVA/VIS-induced DNA damage (Table 2). Importantly, the study by Malhomme de la Roche H, *et al.* clearly demonstrated inter-individual variability in the effect, classifying the participants into two groups: responders and non-responders. However, the molecular mechanisms underlying this phenomenon of inter-individual variabilities remain to be studied in detail.

## Other

In addition to anthocyanin-rich food and beverages, coffee and green tea, we also identified *other* foods and beverages, such as apples, both organic and conventional^(62)^, meal rich in flavonols^(42)^, blood orange juice^(61)^ or soya milk^(63)^, as well as one plant extract*, i.e.* resveratrol-containing food supplement^(64)^, that were studied in different human intervention studies for their protective effects against DNA damage. Two of these studies were acute designs^(61,62)^, while the others were conducted over periods ranging from 5 days to 4 weeks. Four out of the five studies included healthy non-smokers, with one study investigating the effects of dietary flavonols against oxidative DNA damage in patients with type 2 diabetes^(42)^. Regarding the sex of the participants, one study included only females^(61)^, two studies included only males^(62,63)^ and the remaining studies included both males and females^(42,64)^. Nutritionally relevant doses were used in all studies, but only two studies were adequately controlled^(61,63)^. Other assays of genotoxicity were not conducted in none of the studies. However, four out of the five studies evaluated different parameters of oxidative stress/redox status, but none of these parameters was significantly modulated.

Regarding the outcomes of the Comet assay, four out of the five studies reported significant improvements in various parameters of DNA damage, such as increased resistance to H_2_O_2_-induced DNA damage^(61)^, significant decrease in the level of endonuclease III-sensitive sites and an increased capacity to protect DNA against FeCl_3_-induced damage^(62)^, decreased H_2_O_2_-induced DNA damage^(42)^ or progressive decrease in oxidised pyrimidines^(63)^. The reduction in DNA damage reported in these studies ranges from 13 to 74 % (Table 2). One study, however, reported non-significant effects of (poly)phenols on oxidative DNA damage^(64)^.

## Comet assay across the studies

The authors of the studies included in this review employed various modifications of the Comet assay. The predominant, if not exclusive, general type is the alkaline Comet assay, which is conducted with a highly alkaline buffer (300 mM NaOH, 1 mM ethylenediaminetetraacetic acid and pH > 13). Notably, some authors did not report the pH of the buffer, instead directing the readers to previous publications for detailed protocols^(53,64)^. However, this practice often fails to provide the necessary level of detail, which makes it difficult to assess and understand the effects of (poly)phenols accurately and importantly makes it impossible to reproduce experiments.

Using their standard protocols as a basis for their experiments, some of the authors incorporated restriction enzymes into their assays, specifically Fpg and/or endonuclease III, to quantify the levels of oxidised purines and pyrimidines, respectively. The results of these analyses varied across studies, with the levels of oxidised bases being either significantly modulated or non-significantly affected, largely depending on the study design, type and duration of the intervention and the population studied. Additionally, a version of the Comet assay specifically designed to assess H_2_O_2_-induced DNA damage was used in many studies to evaluate DNA resistance to oxidative stress. Again, the results varied across studies, ranging from highly significant to non-significant, depending on the factors mentioned above, and presumably also due to the numerous minor modifications of the general Comet assay protocol across laboratories.

Other types of induced DNA damage, as well as the resistance to them, assessed using the Comet assay across studies eligible for inclusion in this review, include: (+/–)-anti-benzo[*a*]pyrene-7,8-dihydrodiol-9,10-epoxide-induced DNA-damage^(52)^, 3-amino-1-methyl-5H-pyrido[4,3-*b*]indole acetate-induced DNA-damage^(53)^, irradiation-induced DNA damage^(59,60)^ or FeCl_3_-induced DNA damage^(62)^.

Notably, all studies included in this review employed blood cells for conducting the Comet assay.

## Discussion

To our knowledge, this study is the first to systematically review data on the impact of (poly)phenol-rich dietary sources on DNA damage in human intervention studies using the Comet assay. The study is focused on nutritionally relevant dietary sources of (poly)phenols and quantities relevant to human nutrition. Since medicinal plants are primarily used for pharmaceutical purposes and administered in pharmacological doses, they were not considered for inclusion in our study. Additionally, to eliminate the influence of growth (in children and adolescents) or advanced age (in individuals over 70 years of age) on the impact of dietary (poly)phenols on DNA damage, we focused our study on the adult population aged between 18 and 70 years. The findings clearly demonstrate the protective effects of (poly)phenols against DNA damage in humans. Specifically, the majority of the studies reported significant improvements in at least one measure of DNA damage, with three studies showing non-significant results, and only one study indicating a potential adverse effect. While the number of eligible studies was limited, the available data provided a meaningful overview of the current state of research in this field.

The majority of the eligible studies were conducted using (poly)phenol-rich foods and beverages, while only one utilised a plant extract in the form of a food supplement. Notably, (poly)phenol-rich foods and beverages contain various macro- and micronutrients, as well as other bioactive compounds, which may contribute to protection against DNA damage, particularly when the intervention is not adequately placebo controlled. Additionally, most of the studies included only healthy subjects, despite the evidence that individuals with certain diseases are more likely to show positive outcomes, as it has been previously reported^(71)^.

Interestingly, using our search criteria, we did not identify any human intervention studies on dietary (poly)phenols and DNA damage assessment using the Comet assay published after 2016. However, the Comet assay has continued to be employed in several studies on human nutrition since then. For example, it has been reported that the mean level of DNA damage is nearly twice as high in obese women compared with non-obese women, and that vitamins C and E are inversely associated with the level of DNA damage^(72)^. Additionally, studies have shown that blood concentrations of long-chain omega-3 fatty acids, EPA and DHA are inversely associated with DNA damage in Brazilian children and adolescents^(73,74)^ and that a pescatarian diet may be more beneficial for maintaining DNA integrity compared with vegetarian dietary pattern^(75)^.

It is of note that our literature search revealed that only a limited number of (poly)phenol-rich dietary sources have been studied for their effects on DNA damage in human intervention studies using the Comet assay, highlighting the need for further research in this area.

Regarding the molecular mechanisms of action of (poly)phenols on DNA damage, studies have primarily focused on their effects on oxidative stress and cellular antioxidant systems. Accordingly, in most of the studies included in this review, biochemical markers indicating the levels of oxidative stress and/or antioxidant defence were measured, with many demonstrating a statistically significant beneficial effect. In this context, a recent *in vitro* study clearly demonstrated the influence of colonic-microbiota-derived phenolic catabolites on the expression of the *NRF2* transcription factor, the master regulator of redox homeostasis^(76)^. Under normal conditions, NRF2, located in the cytosol, is associated with Kelch-like ECH-associated protein 1, which assists in the ubiquitination of NRF2. In cases of mild oxidative stress, Kelch-like ECH-associated protein 1, functioning as a redox sensor, allows newly synthetized NRF2 molecules to escape ubiquitination, migrate into the nucleus and activate the transcription of target genes by binding to the antioxidant response element in their promoter region^(77)^, thus enhancing the cellular antioxidant defence. Notably, this *in vitro* study^(76)^ was conducted using colon-derived (poly)phenol metabolites at physiologically relevant concentrations, thus providing valid experimental evidence for the biological effects of (poly)phenols.

On the other hand, another experimental study showed that the topical application of apigenin, a flavonoid from the class of flavones, reduces the generation of reactive oxygen species in the skin of mice exposed to ultraviolet B irradiation. This effect was accompanied by a reduction in DNA damage, mediated by the induction of genes involved in the rapid repair of damaged DNA, which represents an important molecular mechanism of action of apigenin on DNA damage. Simultaneously, the study demonstrated decreased expression of the NF-*k*B protein, a key redox-sensitive and pro-inflammatory transcription factor and a major mediator of inflammation, highlighting another potential mechanism of action^(78)^. However, given the chemical diversity of (poly)phenols, their extensive metabolism in the human body and the current advancements in the in the field of (poly)phenols and health, such as the use of multi-omics technologies and advanced bioinformatic methods, it can be expected that future in-depth and comprehensive studies will uncover new, still unexplored mechanisms of action of (poly)phenols on DNA damage.

Through our literature search, along with the selection of eligible studies and data extraction, we gained valuable insights into the use of the Comet assay in human intervention studies with dietary (poly)phenols. Notably, our findings highlight significant inter-laboratory variations in the types of the Comet assay employed across the eligible human intervention studies, such as the alkaline Comet assay, the Comet assay with Fpg enzyme, the Comet assay with endonuclease III, the Comet assay upon H_2_O_2_-induced DNA damage and others. Moreover, some of the measures of DNA damage were significantly modulated in some studies, while remaining constant in others. In addition to differences in interventions and study populations, variations in Comet assay protocols across laboratories may have contributed to these discrepancies. However, it is difficult to identify the conditions and protocols specific to each of the eligible studies, as detailed Comet assay procedures are not always fully described. Over the years, significant efforts have been made to standardise the Comet assay^(79)^. A Consensus Statement for the Minimum Information for Reporting Comet Assay was proposed, providing recommendations for describing Comet assay conditions and results. Adherence to Minimum Information for Reporting Comet Assay recommendations should ensure that Comet assay results can be easily interpreted and independently verified by other researchers^(80)^.

Modifications in recent years have made the Comet assay less time-consuming and less labour-intensive. Flash comet is a modification in which LiOH is used instead of NaOH during unwinding and electrophoresis. This allows for a reduction of the unwinding time from 40 min to 2·5 min, a reduction in the electrophoresis time from 20 min to 1 min and the use of a higher voltage during electrophoresis (5 V/cm instead of 0·7 V/cm)^(81)^. The CometChip assay is a modification that allows running of different samples in a 96-well format, thus increasing Comet assay throughput and reproducibility^(82,83)^. Additionally, fully automated image analysis systems have recently been developed, featuring automatic selection and focusing of Comets, which allows much faster scoring. While analysing 100 samples using ‘manual’ methods might take 1 or 2 days, an automated system can complete the same analysis in 2–4 h. Besides increased speed, automated systems also provide unbiased analysis, free from subjective selection by the researcher^(22)^. An additional aspect to consider in Comet assay analysis is the shape of the comet, which can differentiate between random, double-strand and single-strand DNA breaks^(84)^.

Although we did not find recent evidence of its use in human intervention studies with (poly)phenols, we believe that the Comet assay remains a viable method, suitable for future studies on (poly)phenols and their beneficial health effects in humans, especially given recent improvements in standardisation and automation. In the future, it will be of particular interest to analyse as many different aspects of the Comets as possible^(84)^, which may lead to new insights into the DNA-protective properties of (poly)phenols. These data could be integrated with other analysed parameters, such as phenotypic improvements and modulations at the levels of the metabolome, transcriptome, proteome and gut microbiome. Ideally, all these data could be incorporated into machine learning algorithms^(35)^, with the ultimate goal of gaining a deeper understanding of the health-promoting properties of dietary (poly)phenols in humans.

Our study has some limitations, such as the small number of broad terms used in the literature search, which may have led to the omission of certain studies. Additionally, the literature search was conducted solely in PubMed due to a lack of access to databases such as Web of Science and Scopus. However, we believe that this work represents a comprehensive overview and provides sufficient evidence on the current state of the art of human intervention studies investigating (poly)phenols and the Comet assay. We see this evidence as having the potential to serve as a foundation for developing perspectives on designing future studies in this field.

In conclusion, given the significant technological advances in the performance of the Comet assay, it remains a viable and relevant method for use in human intervention studies examining the protective health properties of (poly)phenols. The Comet assay has the capacity to be integrated into the protocols of future human intervention studies, alongside other standard and advanced analytical methods, including omics and integrative multi-omics approaches.

## Supporting information

Milev et al. supplementary material 1 Milev et al. supplementary material

Milev et al. supplementary material 2 Milev et al. supplementary material

## Supplementary material

For supplementary material/s referred to in this article, please visit https://doi.org/10.1017/S000711452500073X
