# Supplementary material for: Impact of (poly)phenol-rich dietary sources on DNA damage: insights from human intervention studies using the Comet assay – a review and perspective
Source: Br J Nutr. 2025 Apr 7;134(1):1–15. doi: 10.1017/S000711452500073X (PMC12379115; doi:10.1017/S000711452500073X)
Supplement: Milev et al. supplementary material 1 — Milev et al. supplementary material [file S000711452500073Xsup001.docx]

**Illustrations used for preparing the Graphical Abstract**

1. NIH BIOART source
   1. Scientist – Grey

<https://bioart.niaid.nih.gov/bioart/485>

- 1. Human Male Outline Type IV

<https://bioart.niaid.nih.gov/bioart/236>

- 1. 96 Well Plate

<https://bioart.niaid.nih.gov/bioart/7>

- 1. Schematic Glycoprotein - Yellow

<https://bioart.niaid.nih.gov/bioart/468>

- 1. DNA

<https://bioart.niaid.nih.gov/bioart/124>

- 1. RNABrush

<https://bioart.niaid.nih.gov/bioart/453>

- 1. Glutamate

<https://bioart.niaid.nih.gov/bioart/174>

- 1. Microbiota - Beige

<https://bioart.niaid.nih.gov/bioart/349>

- 1. Man Entering Data

<https://bioart.niaid.nih.gov/bioart/333>

1. FREEP!K
   1. Microscope laboratory device

<https://www.freepik.com/free-vector/microscope-laboratory-device_145148788.htm#fromView=keyword&page=2&position=10&uuid=18c47213-e24a-4bd4-85e9-18a564642f45>

- 1. Ripe Berries Template

<https://www.freepik.com/free-vector/ripe-berries-template_3998752.htm#fromView=search&page=1&position=17&uuid=d6b1d468-069f-4402-ab0a-70580f1fe788>

- 1. Close up on coffee mug isolated

<https://www.freepik.com/free-psd/close-up-coffee-mug-isolated_336469200.htm#fromView=keyword&page=1&position=18&uuid=f97a03ca-344d-4c07-a0c7-53c9d38ae571>

- 1. Watercolor orange

<https://www.freepik.com/free-vector/watercolor-orange_1008402.htm#fromView=keyword&page=1&position=6&uuid=5a60d42e-987c-4563-aaaf-ecd42ecdc494>

- 1. Realistic apple top view set. 3d ripe fruit sliced half, whole and green leaves.

<https://www.freepik.com/free-vector/realistic-apple-top-view-set-3d-ripe-fruit-sliced-half-whole-green-leaves_2890874.htm#fromView=keyword&page=1&position=26&uuid=7913f0ac-6617-4d8f-b4b1-1fc550ea5f89>
